# Regulating COX10-AS1 / miR-142-5p / PAICS axis inhibits the proliferation of non-small cell lung cancer

**DOI:** 10.1080/21655979.2021.1957072

**Published:** 2021-07-29

**Authors:** Xinyu Tang, YiHe Wu, Jie Yang, Wenyu Zhu

**Affiliations:** a Department of Oncology, Ruijin Hospital Wuxi Branch, Shanghai Jiao Tong University School of Medicine, Wuxi, China; b Department of oncology, The People's Hospital of Rugao, Jiangsu, Rugao, China; c Department of Thoracic Surgery, the First Affiliated Hospital, Zhejiang University School of Medicine, Zhejiang, China; d Department of Clinical medicine, Yangzhou University’s Medical Faculty, Jiangsu, China; e Department of Oncology, The Affiliated Changzhou No.2 People's Hospital with Nanjing Medical University, Jiangsu, Changzhou, China

**Keywords:** Non-small cell lung cancer (NSCLC), COX10-AS1, MIR-142-5P, PAICS, glycolysis

## Abstract

Non-small cell lung cancer (NSCLC) is one of the main causes of death in the world. To improve the diagnostic level and find new biological targets，GSE datasets were selected from GEO databaseto analyze the differential expression genes and construct ceRNA network. Cell apoptosis detection showed that both the early and late apoptosis rates were increased after inhibition of COX10-AS1. Glycolysis cell-based assay also found that the content of L-lactate decreased significantly after using miR-142-5p mimics but increased after using si-COX10-AS1. Dual-luciferase reporter analysis showed that the luciferase activity of PAICS-WT reporter vector was inhibited by miR-142-5p mimics, but there was no significant change in PAICS-MUT reporter vector after transfection of miR-142-5p mimics. And overexpression of miR-142-5p reduced the level of PAICS, but inhibition of miR-142-5p expression increased the expression of PAICS. After using COX10-AS1, the expression of PAICS inhibited by miR-142-5p was restored. Through bioinformatics analysis, we constructed the COX10-AS1/miR-142-5p/PAICS axis, which is a ceRNA regulatory network. We confirmed that COX10-AS1 down-expression can restore the inhibitory effect of miR-142-5p on PAICS, promote the apoptosis of NSCLC cells, and inhibit the proliferation of NSCLC cells. This process may be mediated by the activation of glycolysis pathway. The glycolysis-related gene PAICS may be a new and significant target for the regulation of the development of NSCLC.

## Introduction

Lung cancer is one of the most common causes of cancer death worldwide. According to previous study [1], there were about 2.1 million lung cancer cases and 1.8 million people died as a result of lung cancer in 2018 worldwide, and the 5-year survival rate of lung cancer is between 4% and 17%. About 85% of this disease belong to non-small cell lung cancer (NSCLC) [2], which is one of the main causes of global disease death [3]. Most NSCLC patients are advanced in diagnosis [4], so the surgical treatment is not effective, and even if the operation, the recurrence rate is higher. NSCLC has a low overall cure rate and survival rate [5–7], which seriously affects the patients’ quality of life, and also costs a lot of prevention and treatment, which is very burdened to family and society. Therefore, it is very important to improve the diagnosis level and find new and more instructive biological targets.

Feinberg et al. [8] have found the volatile characteristics of cancer cells. This is a new method in cancer research, which is helpful to diagnose tumor quickly and simply in vivo and in vitro, and provides theoretical guidance for the prevention and treatment of cancer. Glycolysis is one of the main factors that cause this volatile signal. In the presence of glucose, cells usually take glucose and convert it into pyruvate in the cytoplasm sol by glycolysis. Under the condition of constant oxygen, pyruvate is further transported to mitochondria, where it is oxidized and phosphorylated through the tricarboxylic acid cycle and electron transport chain to generate energy [9]. Cancer cells metabolize faster than normal cells, so they need more energy. The growth state of cancer cells depends on the energy produced by glycolysis to a large extent, because glycolysis has the advantage of producing energy efficiently, which can provide the basic material for the synthesis of amino acids and fatty acids needed for the proliferation of cancer cells [10]. Glycolysis is an important feature of tumor cells, which provides the main energy source for the rapid expansion of tumor cells [11]. Many scholars have found that NSCLC cells can also use glycolysis to maintain its proliferation and promote NSCLC progress [12,13].

NSCLC is a malignant tumor disease, and its progress is mediated by a variety of regulators including miRNA and lncRNA [14]. MiRNA is an endogenetic and small RNA with a length of about 20–24 nucleotides. It is involved in the post-transcriptional regulation of gene expression in multicellular organisms by affecting the stability and translation of mRNA. Long noncoding RNAs (lncRNAs) are a kind of noncoding RNA with more than 200 nucleotides in length, and are various molecules with various functions. Its abnormal expression characteristics have been widely considered to be related to many tumor-causing processes, such as proliferation, metastasis and anti-apoptosis. lncRNA can reduce miRNA abundance, thus alleviate the inhibition of miRNA on downstream target genes [15,16], and participate in the development and occurrence of tumor. Therefore, this study aims to screen biological targets related to NSCLC through bioinformatics, and provide more support for NSCLC diagnosis and mechanism research.

## Methods

### Bioinformatic analysis

R 4.0.1 was used for data set filtering. With ‘non-small cell lung cancer’ or ‘nonsmall cell lung cancer’ or ‘non small cell lung cancer’ or ‘NSCLC’ as the keywords, we searched from the Gene Expression Omnibus (GEO) database. The screening condition was set as the control between the healthy and diseased tissues in the experimental group. The GSE data sets that met the subject were screened. All the data sets were related to diseases. The expression matrix after log2 standardization was analyzed, and the key differentially expressed genes (DEGs) were obtained by intersection. The screening conditions of DEGs were p < 0.05, | log Fold Change (FC) | > sum (abs (GSE logFC)). The obtained differential genes were analyzed by Gene Ontology (GO) to find out the main pathway of differential gene enrichment. The main genes in the main enrichment pathways were selected for Protein–Protein Interaction (PPI) analysis and core network analysis. Use GEPIA2 for survival analysis.

### Cells culture

Human NSCLC cell line A549 (CL-0016) was purchased from Procell Life Science & Technology Co., Ltd (China). And the cells were cultured in Ham’s F-12k (PM150910, Procell, China) supplemented with 10% fetal bovine serum (FBS, 164,210–500, Procell) and 1% P/S (PB180120, Procell) at 5% CO_2_, 37°C.

### Dual-luciferase reporter assay

The wild-type (WT) or mutant-type (MUT) of PAICS 3ʹ-UTR containing the binding sites with miR-142-5p sequences was amplified and separately cloned into the pmirGLO Vector (HH-LUC-016, Promega, China). A549 cells were co-transfected with the WT or MUT constructed luciferase reporter vectors and miR-142-5p mimics (miR-142-5p) or mimics NC (miR-NC) using Escort™ III Transfection Reagent (L3037-SAMPLE, MERCK, America). Subsequently, Firefly/Renilla Dual Luciferase Assay kit (SCT152, MERCK, America) was utilized to determine the relative luciferase activity.

### Glycolysis cell-based assay

Glycolysis cell-based assay kit (600,450–1, Cayman chemical, America) was used for correlation analysis. Cayman’s glycolysis analysis kit is based on colorimetric method to detect the content of L-lactic acid in culture medium to reflect the state of glycolysis reaction process. Firstly, lactate dehydrogenase oxidizes lactate to form pyruvate and Danh, then NADH reduces it to form color product, and finally the absorbance value is measured at 490 nm. The culture supernatant of each group is collected, and 1000 cells are collected after centrifugation at rpm for 5 min, the supernatant was discarded and added to the buffer solution for resuspension. The supernatant was added to the enzyme plate, and then the working solution was added. After incubation at room temperature for 30 min, the L-lactic acid content was detected to evaluate the glycolysis level.

### RT-qPCR

Total RNA was extracted from cells using Trizol reagent (15,596,026, Invitrogen, America) under the manufacturer’s protocols. The complementary DNA was synthesized using SuperScript™ III First-Strand Synthesis SuperMix (11,752,050, Thermo Fisher Scientific, America). RT-qPCR assay was performed with Thermo Scientific Verso 1-step RT-qPCR Low ROX Kit (AB4106 C, Thermo Fisher Scientific, America). The sequences of primers were displayed: PAICS Forward, 5ʹ-TTGCACCGCAGTGTGAAATG-3ʹ; Reverse, 5ʹ-CCACACATCCTGAACTCCCC-3ʹ; miR-142-5p Forward, 5ʹ-GCCGAGACTAACAGCACTGGAGGGTGT-3ʹ; Reverse, 5ʹ-CTCAACTGGTGTCGTGGA-3ʹ; β-actin Forward, 5ʹ-CCGCGAGTACAACCTTCTTG-3ʹ; Reverse, 5ʹ-CAGTTGGTGACAATGCCGTG-3ʹ.

### Western blot

Proteins were extracted using RIPA lysis buffer (P0013B, Beyotime, China) and quantified by BCA kit (P0012S, Beyotime, China) following the standard protocol. An equal amount of extracts was treated with 10% sodium dodecyl sulfate polyacrylamide gel electrophoresis (SDS-PAGE) and shifted onto a polyvinylidene fluoride (PVDF) membrane (Millipore). Subsequently, the membranes were incubated with PAICS Rabbit pAb (1:1000, A6450, Abclonal, China) and GADPH (1:1000, A19056, Abclonal) at 4°C overnight, and followed by interaction with HRP Goat Anti-Rabbit IgG (1:5000, AS014, Abclonal). Finally, protein signals were examined using an ECL method.

### Statistical analysis

All statistical analyses were performed using SPSS 26.0. Quantitative data were expressed as mean ± standard deviation (SD). Student’s *t*-test was used to evaluate difference between the two groups. P < 0.05 was considered statistically significant.

## Results

The DEGs were obtained by differential expression analysis, and the data were cleaned and gene screened by R to construct the ceRNA network.

### Dataset filtering results

**Figure 1.**
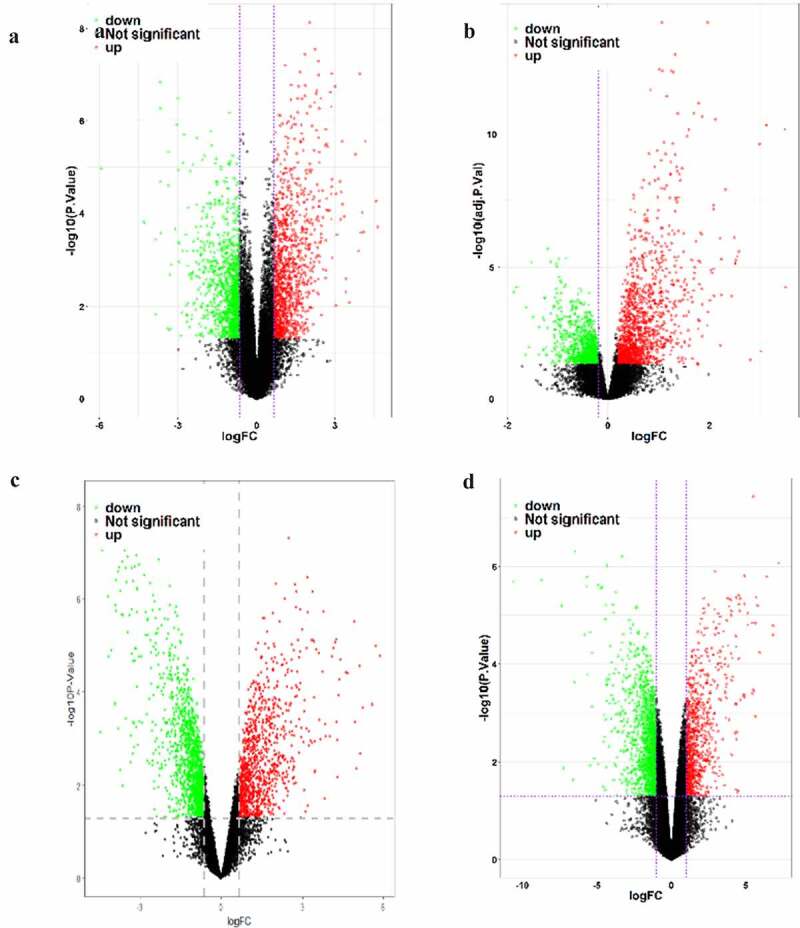
Map of differentially expressed genes in four mRNA datasets. GSE136043 data set (a), GSE103512 data set (b), GSE98929 data set (c), GSE146460 data set (d), in which the red dots are up-regulated genes and the green dots are down-regulated genes

We screened six datasets related to NSCLC from the GEO database (Table 1). Among them, four datasets (GSE136043, GSE103512, GSE98929 and GSE146460) were screened for differentially expressed mRNAs in NSCLC versus normal tissues (Table 2 and Figure 1a-d).

**Table 1.** Basic information of data set

Data sets	Sample capacity (Tumor)	Sample capacity (Nomal)	GPL information	Sample type
GSE98929	5	5	GPL16956 Agilent-045997 Arraystar human lncRNA microarray V3 (Probe Name Version)	mRNA
GSE103512	60	9	Affymetrix HT HG-U133+ PM Array Plate	mRNA
GSE146460	3	3	GPL20115 Agilent-067406 Human CBC lncRNA mRN Amicroarray V4.0 (Probe name version)	mRNA
GSE136043	5	5	GPL13497 Agilent-026652 Whole Human Genome Microarray 4x44K v2 (Probe Name version)	mRNA
GSE137445	4	8	GPL21827 Agilent-079487 Arraystar Human lncRNA microarray V4 (Probe Name version)	lncRNA
GSE102286	91	88	GPL23871 NanoString nCounter Human miRNA Expression Assay v1.6	miRNA

**Table 2.** Screening results of differentially expressed genes

Data sets	Screening criteria	UP	DOWN
GSE136043	|logFC|> 0.656644627259147	1214	1129
GSE98929	|logFC|> 0.625700309380472	1129	1442
GSE103512	|logFC|> 0.19125475620683	1379	1040
GSE146460	|logFC|> 1.00859339012076	839	1578

UP: Number of differentially expressed up-regulated genes. Down: Number of differentially expressed down-regulated genes.

### Analysis of core gene expression

**Figure 2.**
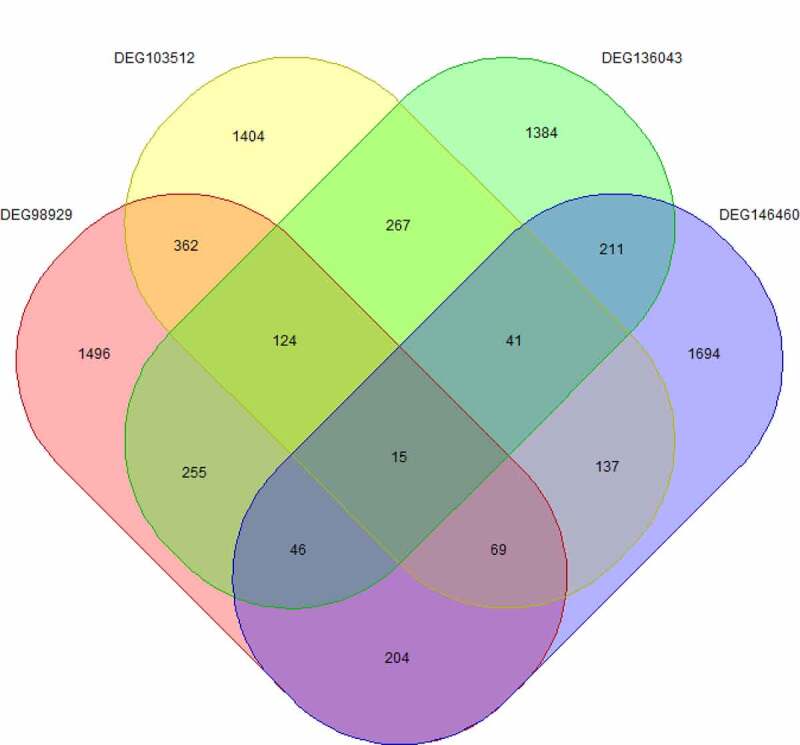
Cross analysis of differentially expressed genes

Fifteen core genes (Figure 2) were obtained by cross analysis of DEGs in four datasets and showed as follows: CDKN3, MND1, OTUD1, SYNC, TOP2A, HN1, BDH2, ADARB1, PIP5K1B, PAICS, LAMP3, KAL1, SCGB3A1, UQCRH and DNAJA3 (Table 3).

**Table 3.** LogFC value of core gene

Gene	DEG146460_logFC	DEG136043_logFC	DEG103512_logFC	DEG98929_logFC
CDKN3	−1.079565303	−0.894571231	−0.938190682	−3.29512032
MND1	1.764380985	−1.370187168	−0.407432851	−2.97776092
OTUD1	−1.632397685	0.82821586	0.603980102	2.01120286
SYNC	−1.170364063	1.240823194	0.332259769	2.60230576
TOP2A	−1.220762	−0.952645586	−1.877043822	−4.1844927
HN1	1.096003529	−0.82824319	−0.911629734	−1.2361343
BDH2	1.056673077	0.788972634	0.6783624	1.2802778
ADARB1	−1.400983387	1.437982898	0.631936113	2.19577292
PIP5K1B	−3.029499639	1.40373747	0.369277616	2.4828686
PAICS	−1.171565814	−0.875196096	−1.031149587	−2.7630676
LAMP3	−4.152279757	1.716864582	1.294265967	3.62350028
KAL1	−3.115241098	2.2683059	0.464148552	1.4088892
SCGB3A1	−2.995107578	−3.277011361	1.595456089	2.67161228
UQCRH	−1.741143902	−0.782546485	−0.538876693	−0.6391598
DNAJA3	−1.47619	−1.33674	−0.44482	−1.0463

### Pathway enrichment analysis and construction of co-expression network

**Figure 3.**
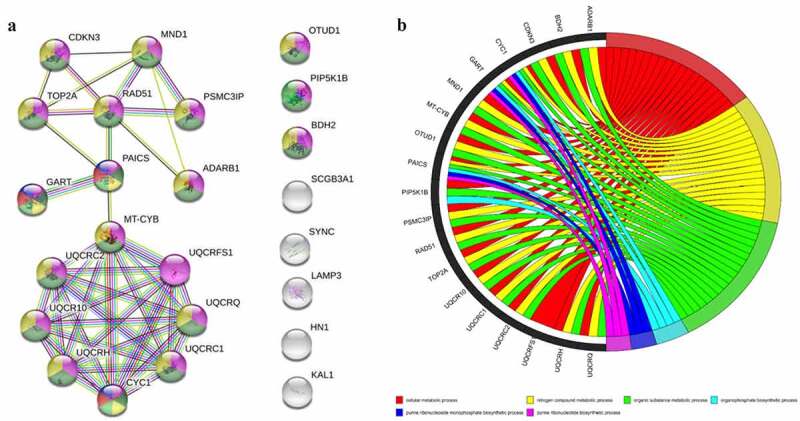
Enrichment analysis of differentially expressed gene pathways and PPI network. (a): PPI network of core genes. (b): General set enriches chord graph. PPI, Protein–Protein Interaction

The protein–protein interaction network was constructed by using STRING database for pathway enrichment analysis. The interaction between the 15 core genes was not statistically significant. The results of PPI interaction network analysis are as follows: number of nodes: 24, number of edges: 42, average node degree: 3.5, avg. local clustering coefficient: 0.562, expected number of edges: 15, PPI enrichment p-value: 1.76e-08. The results show that the main core modules of these 15 genes are mainly enriched in ‘nitrogen compound metabolic process, purine ribonucleotide biosynthetic process, purine ribonucleoside monophosphate biosynthetic process, cellular metabolic process, organic substance metabolic process, organophosphate biosynthetic process’ pathways, and the corresponding genes are shown in Figure 3a-b.

### Construction of ceRNA network

**Figure 4.**
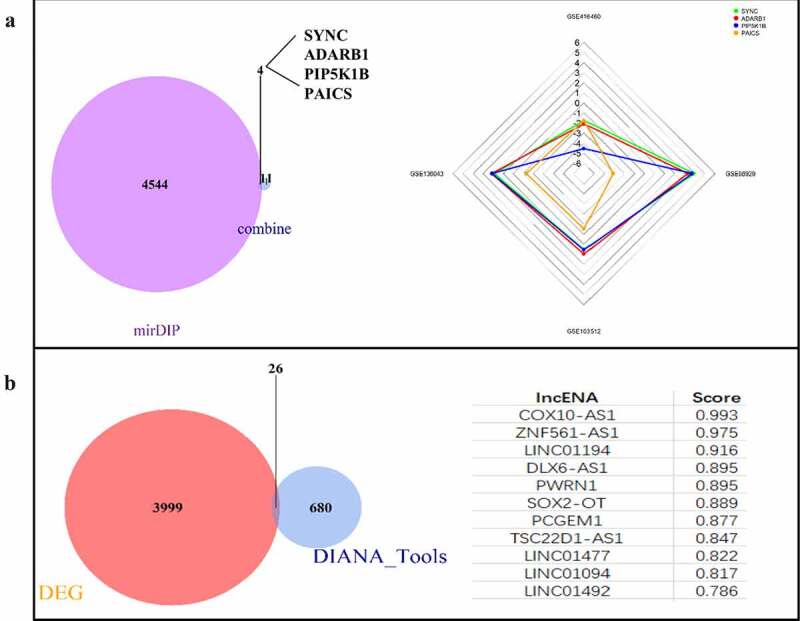
Construction of ceRNA network. (a): mRNA targets predicted from miRNAs. (b): Prediction of lncRNA associated miRNA

MiRNA-GSE102286 data set | logFC| > 0.638066089552239, 55 DEGs were screened, including 18 up-regulated genes and 37 down regulated genes. The 55 differentially expressed miRNAs were predicted by online website mirdip, and 4548 mRNAs were predicted. At the same time, they intersected with 15 core genes. The results showed that four genes, SYNC, ADARB1, PIP5K1B and PAICS, were obtained, among which PAICS had the most significant difference, and PAICS was the target gene of hsa-miR-142-5p (Figure 4a). According to the lncRNA-GSE137445 data set | logFC | > 0.461260700252269, 4025 DEGs were screened, including 2361 up-regulated genes and 1664 down regulated genes. Using the online website Diana tools lncbase predicted v. 2. A total of 706 lncRNAs corresponding to hsa-miR-142-5p were predicted and crossed with lncRNA-GSE137445 data set to obtain 26 intersections: COX10-AS1, znf561-as1, linc01194, dlx6-as1, pwrn1, sox2-ot, pcgem1, tsc22d1-as1, linc01477, linc01094, linc01492, linc00943, st3gal6-as1, linc00907, trhde-as1, pax8-as1, hoxc-as, ptprd-as2, zranb2-as2, Linc01010, slc26a4-as1, snhg14, ckmt2-as1, linc00346, hcg18, nutm2b-as1. The highest score of lncRNA predicted by Diana tools lncbase predicted v.2 was COX10-AS1 (Figure 4b). According to the online network prediction results, we constructed a ceRNA network, namely COX10-AS1/miR-142-5p/PAICS.

### Sensitivity analysis of target genes

**Figure 5.**
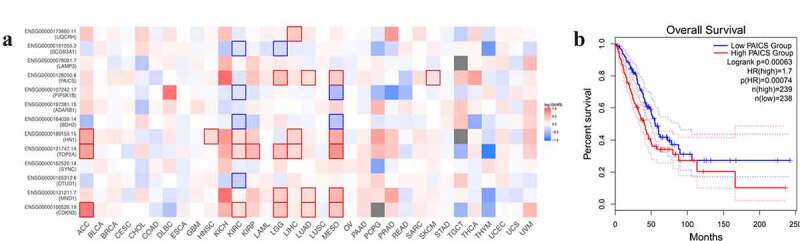
Sensitivity analysis of PAICS. (a): Survival contribution of multiple genes in multiple cancer types. (b): The impact of PAICS on the survival of LUAD

Mantel-Cox test was used to estimate the survival contribution of 15 core genes in various cancer types. KAl1 data is not shown in the figure due to database reasons. It can be seen from the figure that PAICS has a great influence on the survival rate of lung adenocarcinoma (LUAD) (Figure 5a). According to the expression of PAICS, the survival analysis and Kaplan Meier curve showed that the high expression of PAICS had a poor prognosis for LUAD. The HR (hazard ratio) of PAICS high expression group/low expression group was 1.7, which indicated that the death rate of PAICS high expression group was faster than that of PAICS low expression group. Log rank test showed that there was significant difference between the two groups (P = 0.00063) (Figure 5b).

### Research on molecular mechanism

**Figure 6.**
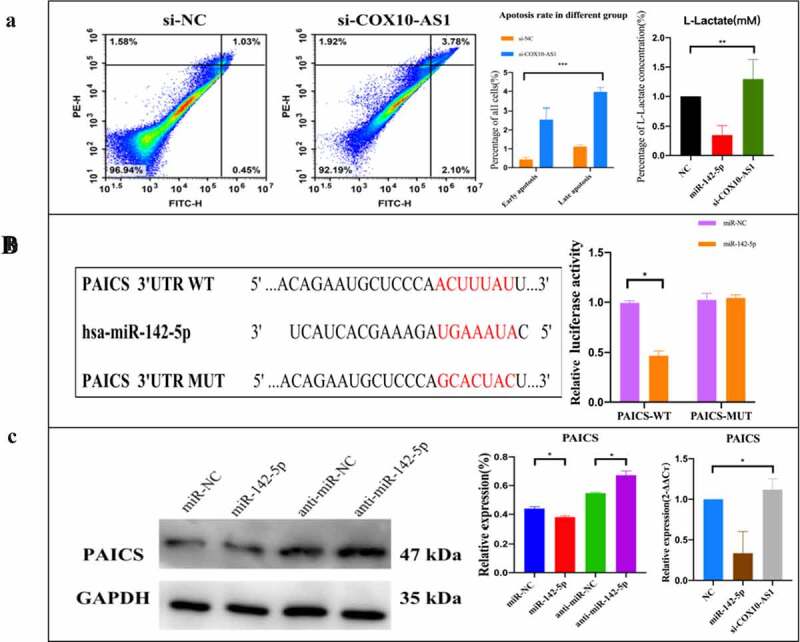
The effect of COX10-AS1/miR-142-5p/PAICS axis on the proliferation of NSCLC cells. (a): Annexin V-FITC/PI apoptosis detection and lactic acid content detection. (b): miR-142-5p/ PAICS interaction was detected. (c): Regulation of COX10-AS1/miR-142-5p on PAICS gene and protein expression

To clarify the potential biological function of COX10-AS1/miR-142-5p/PAICS in NSCLC, annexin V FITC/PI kit was used to detect cell apoptosis. The results showed that the early and late apoptosis rates increased after inhibition of COX10-AS1. Glycolysis cell-based assay also showed that the level of L-lactate decreased significantly after using miR-142-5p mimics but increased after using si-COX10-AS1 (Figure 6a). In order to study the biological pathway of miR-142-5p/PAICS in the pathogenesis of NSCLC, Starbase was used to predict the binding site of PAICS and miR-142-5p. After double luciferase reporter analysis, it was found that the luciferase activity of PAICS-wt reporter vector was inhibited by miR-142-5p mimics, but there was no significant change in PAICS-mut reporter vector after transfection of miR-142-5p mimics (Figure 6b). Subsequently, Western blot and QRT PCR analysis showed that miR-142-5p overexpression decreased the level of PAICS, but inhibition of miR-142-5p expression increased the expression of PAICS. After using COX10-AS1, the expression level of PAICS inhibited by miR-142-5p was restored (Figure 6c). These results suggest that PAICS can interact with miR-142-5p, and COX10-AS1 can affect the biological effect of miR-142-5p.

## Discussion

The incidence rate of NSCLC is high, and the 5-year survival rate remains low. One of the reasons is that most NSCLC patients are in the middle and late stages when they are discovered. Therefore, it is of great significance to explore the biological targets of NSCLS. Glycolysis, as a metabolic process occurring in the process of tumor formation, allows cancer cell glycolysis intermediates to enter purine and pyrimidine biosynthesis pathways to maintain and increase cancer cell proliferation [17,18]. Therefore, inhibition of glycolysis is considered as a treatment for invasive cancer including lung cancer. Recently, it has been found that glycolysis and gluconeogenesis are activated in NSCLC in the way of tumor size and oxygenation regulation and have different correlation with prognosis [19].

In recent years, bioinformatics technology has been introduced into the field of medical molecular biology, which has rapidly expanded the scope of basic research and enriched the research content of biological targets [20,21]. Therefore, with the help of bioinformatics, through the comprehensive mining and analysis of four mRNA sets, one miRNA data set and one lncRNA data set in NCBI database, the data mining based on high-throughput data analysis technology improves the screening accuracy of DEGs. Fifteen genes with research value were screened from 9126 DEGs. Then, pathway enrichment analysis combined with miRNA and lncRNA information added, the regulatory axis of ceRNA was constructed, and COX10-AS1/ hsa-miR-142-5p/PAICS was used as the main index of this study.

PAICS, phosphoribosylamino imidazole carboxylase and phosphoribosylamino imidazole succinocarboxamide synthase, also known as ADE2, which encodes a bifunctional enzyme with N-terminal phosphoramidoimidazolyl carboxylase activity and C-terminal phosphoramidoimidazolyl dicarboxylate amide synthetase, catalyzes the sixth and seventh steps of purine biosynthesis. It encodes a multifunctional protein (ADE2). Studies have confirmed that ADE2 knockout is related to purine biosynthesis, sleep regulation and energy storage [22]. ADE2 enzyme uses air, ATP and HCO_3_ as substrates to participate in the regulation of primary metabolism and can be used as a new intracellular target for the discovery of antifungal drugs [23]. PAICS and its encoded proteins play an important role in new purine biosynthesis pathways, regulating the synthesis and decomposition of glycolysis intermediates. Glycolysis is a process in which glucose or glycogen is decomposed into pyruvate or lactic acid under the condition of oxygen deficiency, accompanied by the production of a small amount of ATP. This process is carried out in the cytoplasm without oxygen, and each reaction step is basically catalyzed by a specific enzyme. Under the condition of hypoxia, pyruvate can receive hydrogen from the dephosphorylation of pyruvate under the catalysis of lactate dehydrogenase, there is some evidence that the level of glucose metabolism in NSCLC tumor is higher than that in adjacent tissues. The extensive use of FDG-PET has shown that many tumors have enhanced glucose uptake in vivo. Changes in gene levels, including KRAS, PIK3CA, LKB1, TP53, enhanced glycolysis flux, indicating that reprogramming glucose metabolism is the common and cell-independent result of these mutations [24,25]. It has been reported in the literature that the metabolism between tumor and benign lung was compared by intraoperative ^13^C-glucose infusion in nine patients with NSCLC. It was observed that lactic acid was used as a potential carbon source for glucose metabolism in NSCLC tumor [26]. At present, some views believe that glucose is preferentially converted to lactic acid under Warburg effect, which inhibits glucose oxidation and lactic acid secretion into extracellular space. However, some literatures have shown that in human NSCLC, compared with the adjacent lung, glucose oxidation is activated [27], and after lactic acid is absorbed, it mainly enters the TCA cycle in the form of energy supply [28]. In this study, lactic acid was selected as a measure of glycolysis reaction of NSCLC. The results of glycolysis cell-based assay also verified that the lactic acid content increased after the expression of PAICS was up-regulated, suggesting that the up-regulated expression of PAICS may promote the glycolysis process, provide intermediate products for the proliferation of NSCLC cells, and play a role in promoting cancer, this is consistent with the results of PAICS overexpression in lung adenocarcinoma [29]. Pathway enrichment analysis also showed that PAICS was involved in purine nucleoside biosynthesis, and purine nucleoside monophosphate biosynthesis played a role in cell metabolism and organic matter metabolism [30]. Therefore, it can be speculated that PAICS regulates the level of lactic acid and affects the glycolysis process, which may provide a possible strategy for the prevention and treatment of NSCLC.

MiR-142 is the first microRNA isolated from mouse hematopoietic tissue. It is enriched in adult hematopoietic tissue and distributed in bone marrow, spleen, liver and fetal liver. miR-142-5p is one of the two mature forms of mir-142 encoded by the classical stem ring structure. It is very conservative in human, rat and mouse [31–34]. It was found that the expression of miR-142-5p was up-regulated in atherosclerotic plaques in apolipoprotein E deficient mice. In human macrophages stimulated by ox-LDL, miR-142-5p expression was also up-regulated, while inhibition of miR-142-5p resulted in decreased macrophage apoptosis, which may be through targeting transforming growth factor β [35]. In the pig model of autologous lung transplantation, miR-142-5p increased significantly after lung ischemia-reperfusion, suggesting that miR-142-5p is a regulatory factor in the process of lung ischemia-reperfusion injury and participates in the occurrence of organ rejection [36]. It has also been reported that up regulation of miR-142-5p can inhibit the proliferation of NSCLC cells [37]. In this study, up regulation of miR-142-5p inhibits the translation of PAICS mRNA and reduces the expression of PAICS, which is consistent with the function of miR-142-5p involved in apoptosis in previous studies, suggesting that miR-142-5p is related to the functional expression of NSCLC cells.

COX10-AS1 belongs to ncRNA, which is the antisense RNA of COX10. It is mainly distributed in lymph nodes and skin tissues, and also expressed in lung tissues. At present, there are few studies on COX10-AS1, some studies show that it sponged miR-361-5p to enhance ACTG1 expression and accelerated tumorigenesis in glioblastoma [38]. Other studies believe that COX10-AS1 is an lncRNA related to autophagy in glioma prognosis [39], and low expression of COX10-AS1 is associated with low survival rate of breast adenocarcinoma [40]. It is suggested that COX10-AS1 could be used as a new target in the study of tumor mechanism. However, the relationship between COX10-AS1 and NSCLC has not been reported. In this study, we confirmed that COX10-AS1 regulates PAICS expression through sponge miR-142-5p. The results suggest that COX10-AS1 affects the biological effect of miR-142-5p and miR-142-5p interacts with PAICS. The changes of apoptosis and glycolysis efficiency of NSCLC cells are related to the regulation of COX10-AS1/miR-142-5p/PAICS axis, but more specific mechanisms still need to be confirmed by further experiments.

## Conclusions

In this study, we constructed the COX10-AS1/miR-142-5p/PAICS axis through bioinformatics analysis, which is a ceRNA regulatory network. It was confirmed that down regulating the expression of COX10-AS1 promotes the apoptosis of NSCLC cells and inhibits the proliferation of NSCLC cells. This process may be mediated by the activation of glycolysis pathway. The glycolysis-related gene PAICS may be a new and significant target for the regulation of the development of NSCLC.

## Highlights

1. A ceRNA regulatory network, COX10-AS1/miR-142-5p/PAICS, was constructed in NSCLC by bioinformatics analysis.
2. COX10-AS1 down-expression can promote the cells apoptosis and inhibit the proliferation of NSCLC cells.
3. COX10-AS1 mediates the development of NSCLC by regulating the glycolytic pathway.
